# Isolation and Biochemical Characterization of a Glucose Dehydrogenase from a Hay Infusion Metagenome

**DOI:** 10.1371/journal.pone.0085844

**Published:** 2014-01-14

**Authors:** Alexander Basner, Garabed Antranikian

**Affiliations:** Institute of Technical Microbiology, Hamburg University of Technology, Hamburg, Germany; Russian Academy of Sciences, Institute for Biological Instrumentation, Russian Federation

## Abstract

Glucose hydrolyzing enzymes are essential to determine blood glucose level. A high-throughput screening approach was established to identify NAD(P)-dependent glucose dehydrogenases for the application in test stripes and the respective blood glucose meters. In the current report a glucose hydrolyzing enzyme, derived from a metagenomic library by expressing recombinant DNA fragments isolated from hay infusion, was characterized. The recombinant clone showing activity on glucose as substrate exhibited an open reading frame of 987 bp encoding for a peptide of 328 amino acids. The isolated enzyme showed typical sequence motifs of short-chain-dehydrogenases using NAD(P) as a co-factor and had a sequence similarity between 33 and 35% to characterized glucose dehydrogenases from different *Bacillus* species. The identified glucose dehydrogenase gene was expressed in *E. coli*, purified and subsequently characterized. The enzyme, belonging to the superfamily of short-chain dehydrogenases, shows a broad substrate range with a high affinity to glucose, xylose and glucose-6-phosphate. Due to its ability to be strongly associated with its cofactor NAD(P), the enzyme is able to directly transfer electrons from glucose oxidation to external electron acceptors by regenerating the cofactor while being still associated to the protein.

## Introduction

Glucose hydrolyzing enzymes are ubiquitous proteins which are present in all domains of life. They are key enzymes in the form of glucose-6-phosphate dehydrogenases and glucose dehydrogenases of the oxidative pentose phosphate pathway and in the classical Entner-Douderoff-pathway in bacteria respectively [1]. These co-factor-dependent enzymes catalyze the oxidation of glucose to gluconolactone by transferring electrons to an oxidized dinucleotide NAD(P)^+^. The formed gluconate gets phosphorylated and transferred into ribulose 5-phosphate. In this pathway glucose dehydrogenases (EC 1.1.1.47) and glucose-6-phosphate dehydrogenases (EC 1.1.) are key enzymes, and play an important role in anabolism, for the generation of reducing equivalents and in the production of precursors for the synthesis of key cell constituents. Major fields of application of these biocatalysts include enzymatic determination of blood glucose level, co-factor regeneration as well as enzymatic production of gluconic acid [2], [3]. Due to the fact that glucose hydrolyzing enzymes are of great interest for blood glucose monitoring devices enormous efforts have been undertaken to identify novel biocatalysts for further improvements of these systems. The development and permanent improvement of blood glucose meters and their test stripes is the basis for an effective diabetes mellitus treatment. Today, a multitude of different enzyme systems derived from bacterial and fungal microorganisms are available on the market [4]–[6]. Hence, the screening of metagenomic libraries derived from environmental habitats provides a promising source for the identification of novel biocatalysts [7].

Various analyses of metagenomic data sets revealed that NAD(P)^+^-dependent glucose dehydrogenases are produced by different bacterial species in nearly every habitat [8]. Several glucose dehydrogenases from *Bacillus* species have been isolated and characterized since the early 1960s and the crystal structure of the glucose dehydrogenase from *Bacillus megaterium* was determined in 2001 [9], [10]. The evaluation of more than 1000 sequences, combined with structural information, showed that bacterial NAD(P)^+^ dependent glucose dehydrogenases belong to the extended superfamily of short-chain dehydrogenases/reductases (SDR) [11].

The highly diverse superfamily of SDR enzymes consist of ≈250 amino acid residues and catalyze cofactor-dependent oxidation/reduction reactions of great functional diversity including oxidoreductases, epimerases, isomerases and lyases [[12], [13]]. Moreover, within the group of oxidoreductases, SDRs have the ability to act on a broad range of compounds like steroids, aliphatic alcohols and sugars [14]. The occurrence of characteristic sequence motifs at defined positions to each other is the main criterion for SDR-protein classification. The N-terminus is responsible for the co-factor binding in the characteristic Rossmann-fold, while the C-Terminus constitutes the substrate binding. The overall structure of the N-terminal Rossmann-fold is thereby conserved and consists of alternating α-helices and β-strands [15]. The strand topology for binding dinucleotids such as NAD(P)^+^ consists of six parallel β-sheets flanked by α-helices with a relative strand order β3-β2-β1-β4-β5-β6 [16], [17]. Highly conserved sequence motifs for co-factor binding are present within the characteristic Rossmann-fold. The connecting loop between β-strand 1 and α-helix 1 is referred to as the ligand binding loop. Conserved amino acid residues from this loop region and the surrounding β-strand and α-helix form the overall conserved sequence motif V/IXGX_1–2_GXXGXXXG/A for Rossmann-folds, responsible for the binding of FAD or NAD(P) [18]. The elements of the active side of SDR enzymes denoted as “classical” are also conserved [12]. In most cases the active side residues consists of a catalytic tetrade including a Tyr, which is highly conserved in all subfamilies of SDR, as well as a Ser, a Lys and an Asn, arranged in the typical manner Asn-x_n_-Ser-x_n_-Tyr-x_3_-Lys [15], [19], [20].

To assure activity of NAD(P)-dependent enzymes the oxidized form of the co-factor molecule needs to be present. The mechanism of catalysis involves an ordered binding of the oxidized cofactor and glucose, followed by an ordered release of the gluconolactone and the reduced co-factor molecule [21]. In contrast most of the FAD-dependent dehydrogenases bind their co-factor via covalent bindings. As a result, the attached co-factor needs to be recycled by an additional electron acceptor.

For many bacterial and archaeal glucose dehydrogenases xylose and other isomers of glucose are suitable substrates as well. Determining sugar specific stereochemical configurations of hydroxyl groups is common to several characterized enzymes [22], [23]. To date, a bacterial enzyme of the SDR-family showing high activity and affinity to glucose and glucose-6-phosphate has not been described yet.

In this work the isolation and characterization of a novel glucose hydrolyzing SDR enzyme from a metagenomic library derived from a hay infusion is presented. We established an activity based high-throughput screening assay and screened more than 20,000 metagenomic clones by expressing DNA fragments from an environmental sample, using glucose as substrate and specific screening mediators related to test stripes applied in blood glucose meters. The identified enzyme was produced in *E. coli*, purified and subsequently characterized.

## Materials and Methods

### High-throughput Screening of a Metagenomic Library

A mixture containing hay and green grass was collected from a lakeside and mixed with 200 ml water containing sediment from the respective lake. The sample collection from the public lakeside required no specific permission and no endangered or protected species were involved.

Afterwards, the hay infusion was incubated for two weeks at room temperature after which its solid phase was removed and the organisms were harvested from the liquid phase by centrifugation. Total genomic DNA was isolated by resuspending the cells in bacterial lysis buffer 1 (50 mM Tris-HCl, 50 mM EDTA, 0.5% Tween®-20, 0.5% Triton X-100; pH 8.0) containing lysozyme, Proteinase K and RNase A, followed by the addition of bacterial lysis buffer 2 (3 M guanidine-HCl, 20% Tween®-20) and an incubation step at 50°C for 30 min.

Total genomic DNA was isolated using the “genomic-tip 100/G” system (Qiagen) for the lysis of bacteria.

The constructed metagenomic library of a hay infusion in *E. coli* XLOLR/pBK-CMV was automatically transferred from LB (Luria-Bertani)-agar plates to 96 well plates by the colony picking system QPix2 (Molecular Devices, New Milton). Gene expression was performed in auto induction media [24] containing 50 µg/ml kanamycin for at least 18 h at 37°C at 220 rpm. Replicated backup cultures were also cultivated in 96 well plates filled with 200 µl 2x LB (20 g/L tryptone, 10 g/L yeast extract, 7.5 g/L NaCl) containing 50 µg/ml kanamycin. Overnight cell growth was performed at 37°C at 220 rpm. The separation of the cells from the growth medium was done by centrifugation in the 96 well plates. The remaining supernatant was removed by pipetting with a speed of 150 µl/s by a Tecan Freedom Evo robot. Subsequently, cell disruption was performed by a freeze-thaw cycle (−80°C/RT) followed by adding 50 µl of 50 mM HEPES buffer containing 20% (v/v) B-PER®, 0.5% (w/v) lysozyme and 10 U/ml DNaseI at pH 7.0 for 1 h at RT. Glucose dehydrogenase activity was determined spectrophotometrically by the reduction of *p*-nitrosoanilline BM 53.0861. The substrate solution consisted of 0.25 mg/L BM 53.0861 and 75 mM D-glucose in 200 mM citrate buffer at pH 6.0. A sample of 180 µl of the substrate solution was simultaneously given to the 96 wells of the microtiter plate. The kinetics of the crude extracts were determined over a period of 1 h incubation at 37°C compared to a negative control without D-glucose. The formation of phenylendiamine by the reduction of the electron acceptor BM 53.0861 was measured at a wavelength of 620 nm in a safire^2^ microplate reader (Tecan). Crude extracts showing activity towards glucose were identified by a change in absorbance less than the values of the negative control above a defined threshold (ΔA_620_ 0.1). Identified positive candidates from the high-throughput screening were subsequently recultivated from the backup plates in 100 ml LB-medium containing 50 µg/ml kanamycin. Gene expression was induced with 1 mM isopropyl-β-D-thiogalactopyranoside (IPTG) at an optical density of the culture between 0.6 and 0.8 for at least 4 h. Subsequently, the cells were harvested by centrifugation, disrupted using French pressure cell®. The gained crude extracts were reconfirmed again on glucose dehydrogenase activity. Phagemid clones showing reproducible activity on glucose were inoculated in 5 ml LB broth containing 50 µg/ml kanamycin and the recombinant phagemids were isolated, purified and sequenced by primer walking in 5′ and 3′ direction and later assembled. Open reading frames (ORF) were detected by the online tool New England BioLabs® Cutter v. 2.0. Sequence homologies of the detected ORF were identified with the basic local alignment search tool (BLAST) program [25]. Multiple alignments between similar protein sequences were performed with the clustalW program [26]. The secondary structure of proteins was predicted by psipred 3.0 [27].

### Cloning and Heterologous Expression of the Glucose Dehydrogenase in *E. coli*

The coding gene *gdh1E5* of the identified ORF was amplified by PCR using the forward primer Frwd_*Sal*I (5′-AAAGTCGACTTTTACTCAGGAAAATCCTGGT-3′) and the reverse primer Rev_*Hind*III (5′-TTTGAAGCTTTTAGCCTAAATGCTCACCG-3′) with the isolated phagemid as template. The introduced restriction sites *Sal*I (forward) and *Hind*III (reverse) are underlined. PCR was performed with the Phusion®-Polymerase according to the manufacturer’s recommendations. The temperature setup was 2 min at 98°C followed by 35 cycles of 20 s at 98°C, 25 s at 55°C, 30 s at 72°C with a final elongation step at 72°C for 7 min. The amplified DNA-fragment was inserted into the cloning vector pJet1.2/blunt and checked by sequencing at Eurofins MWG Operon. Finally, the *gdh1E5* gene was ligated via the added restriction sites *Sal*I and *Hind*III into the double digested and linearized expression vector pQE80L using T4-Ligase. The ligation mixture was used to transform chemical competent *E. coli* C43 (DE3) cells by heat shock. Positive transformants were inoculated in LB broth containing 100 µg/ml carbenicillin at 37°C until an optical density of 0.6–0.8 at OD 600 nm was reached. Afterwards gene expression was induced by IPTG at 30°C overnight.

### Purification of the Recombinant GDH1E5

12 h after gene expression induction the cells were harvested by centrifugation and resuspended in 50 mM Na-Phosphate buffer (pH 6.0) containing 1,5 M ammonium sulfate for the following purification step. The cells were disrupted by french pressure cell, and centrifuged at 20,000×g for 30 min at 4°C. The resulting supernatant was applied to a preequilibrated column of phenyl sepharose (HiLoad 26/10 Phenyl Sepharose HP; GE Healthcare, Munich) for hydrophobic interaction chromatography (HIC) and eluted from the column with a stepwise decreasing gradient of ammonium sulfate. The recombinant protein-containing fractions were pooled, analyzed by sodium dodecyl sulfate-polyacrylamide gel electrophoresis (SDS-PAGE), concentrated and further purified by size exclusion chromatography (HiLoad 16/60 Superdex 200 prep grade; GE Healthcare, Munich). As a final purification step the pooled fractions resulting from size exclusion chromatography were applied to immobilized metal ion affinity chromatography using Ni^2+^-NTA (“Qia*express* Kit, Qiagen”). The enzyme was eluted from the column with 50 mM phosphate buffer (pH 6.0) containing 250 mM imidazole. Protein concentration was determined by the method of Bradford using bovine serum albumin (BSA) as a standard [28]. The purified protein was dialyzed against 50 mM phosphate buffer (pH 6.0) and the purity was analyzed by SDS-PAGE.

### Electrophoresis and Molecular Mass Determination

SDS-PAGE under denatured conditions was performed by using the Mini-Protean system with 10 and 12% acrylamide gels. The used molecular mass marker was “Unstained Protein Molecular Weight Marker” (Fermentas, 14.4 - to 116 kDa range). The staining of acrylamide gels was done in Coomassie blue.

The molecular weight of proteins under native conditions was determined by size exclusion chromatography (HiLoad 16/60 Superdex 200 prep grade; GE Healthcare, Munich) with 50 mM sodium phosphate buffer (pH 6.0) containing 150 mM NaCl. The column was calibrated with protein standard from GE Healthcare in the range of 44,000 to 440,000 Da by calculating the corresponding K_AV_ values and plotting their K_AV_ values versus the logarithm of their molecular weight. Additionally the molecular mass was determined by native PAGE using the Novex® XCell II™ Mini-Cell with precast Tris-glycine gradient gels (4 to 12%) from Anamed Elektrophoresis. The molecular weight was determined using native protein standard from GE Healthcare in the range of 66,000 Da to 669,000 Da by calculating their corresponding retention factor Rf and plotting the values versus the logarithm of their molecular weight.

### Enzymatic Assays

All the enzyme assays were conducted in triplicates in the presence of a negative control without any substrate. For the detection of relative enzyme activities the redox dye 2,6 dichlorophenolindophenol (DCPIP) was used. The standard DCPIP assay was performed by measuring the time-dependent reduction of 1 mM of the external electron acceptor DCPIP in 50 mM Na-phosphate buffer (pH 6.0) containing 50 mM substrate at an absorbance of 600 nm and 35°C. The molar extinction coefficient for DCPIP at 600 nm and pH 6.0 was experimentally determined to be 12.50 M^−1^ cm^−1^. The determination of pH dependency of enzyme activity was performed in Britton & Robinson universal buffer at the desired pH using the experimentally determined corresponding molar extinction coefficient of DCPIP at 600 nm. Alternatively, absolute enzyme activities were detected by the time-dependent reduction of 0.25 mg/L of the *p*-nitrosoaniline derivate BM 53.0861 in 200 mM citrate buffer (pH 6.0) and 75 mM D-glucose. *p*-nitrosoaniline derivates are a much more common class of electron acceptors used in several test stripes of glucose meters as mediator system for the electron transfer [4]. The used molar extinction coefficient of BM 53.0861 at pH of 6.0 was 30 M^−1^ cm^−1^.

### Steady-state Kinetics

All the measurements were performed at 35°C in 200 mM citrate buffer (pH 6.0) using *p*-nitrosoaniline BM 53.0861 as electron acceptor. For the determination of the steady-state kinetics different substrate concentrations in the range of 0.1–350 mM were tested. The kinetic constants were calculated by nonlinear regression, fitting the detected data to Michaelis-Menten equation.

### Characterization of the Glucose Dehydrogenase

Absorbance spectra were performed with purified enzyme preparations in a wavelength range of λ = 250–600 nm using a scan rate of 300 nm/min. The absorbance data collection interval was set to 0.5 nm.

The substrate specificity of the GDH1E5 was determined with various mono- and disaccharides as well as alcohol sugars. For detecting absolute activities towards different substrates the time-dependent reduction of BM 53.0861 in 200 mM citrate buffer with 75 mM of the corresponding saccharide was determined over a period of 5 min at 35°C. The optimum temperature of the enzyme was assayed with DCPIP as electron acceptor and 75 mM D-glucose as substrate at various temperatures in the range of 5 to 60°C. The thermostability of the enzyme was determined at various temperatures in the range of 30 to 50°C. After preincubation of the enzyme solution at the desired temperature, residual activity was determined at different time points with DCPIP under standard conditions. The effect of additives on enzyme activity was assayed at final concentrations of 1 mM, 1% and 5 mM (v/v, w/v) respectively. The influence of organic solvents on the enzyme activity was determined at a final concentration of 10% (v/v). All the tested additives and detergents were added to the enzyme using 200 mM citrate buffer (pH 6.0) and the mixture was preincubated for 30 min at room temperature. Afterwards enzyme activity was determined with DCPIP in 200 mM citrate buffer (pH 6.0) for 5 min and 35°C. The effect of organic solvents on enzyme activity was assayed at final concentrations of 10% (v/v) using sodium phosphate buffer under equal conditions.

## Results

### High-throughput Screening

In the activity based high-throughput screening approach more than 20,000 recombinant *E. coli* clones, containing metagenomic gene fragments, were automatically transferred to 96 well plates by the QPix2 colony picking system. They were subsequently inoculated, harvested and finally screened. Gene expression was performed in an auto inducing medium in the absence of IPTG. Crude extracts were analyzed in an activity based assay using *p*-nitrosoaniline BM53.0861 as an indicative electron acceptor and D-glucose as a substrate for clones expressing sugar hydrolyzing enzymes. The focus in this screening was the identification of novel dehydrogenases with covalently or strongly attached co-factors, to avoid the external addition of expensive co-factor molecules. Overall, 13 clones were identified in the screening showing significant activities above the defined threshold (ΔA_620 nm_>0.1) and were recultivated in a total volume of 50 ml. Next, the clones were analyzed for glucose dehydrogenase activity again using *p*-nitrosoaniline BM53.0861 as electron acceptor to confirm the corresponding results from the high-throughput screening.

### Sequence Analysis

**Figure 1:**
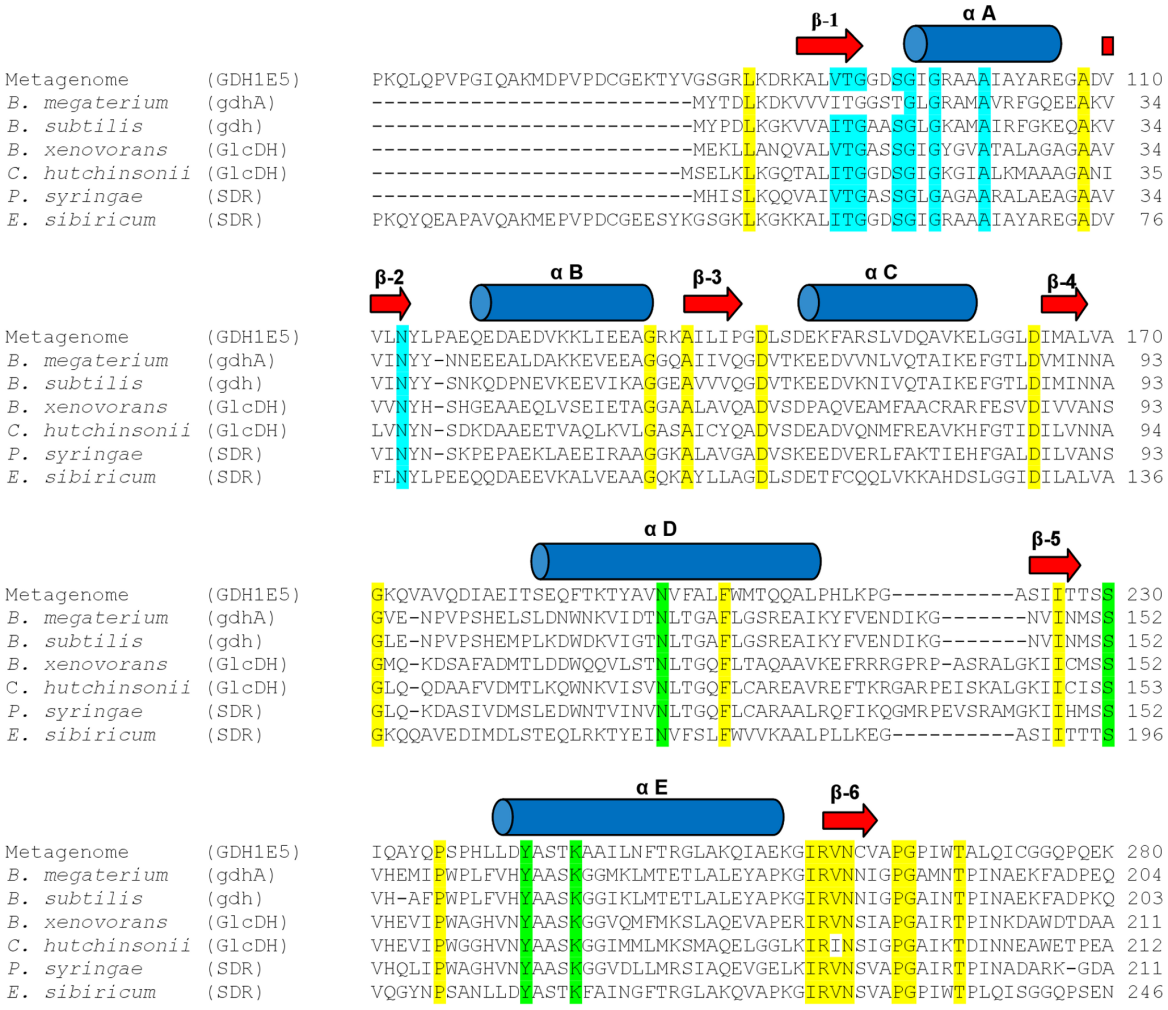
Amino acid sequence alignment of the GDH1E5 with several glucose dehydrogenases and SDR enzymes. The secondary structure of the GDH1E5 (CCK35875) was predicted by the online tool PSIPRED 3.0 and is indicated as arrows for β-strands and as cylinders for α-helices. Conserved structural residues of SDR enzymes are highlighted. Active site residues are colored in green, residues for co-factor binding in blue and conserved structural residues in yellow. The sequence of the GDH1E5 was aligned with Glucose 1-Dehydrogenases (gdhA, gdh, GlcDH) from *Bacillus megaterium* (P10528), *Bacillus subtilis* (P12310), *Burkholderia xenovorans* (YP_554854), *Cytophaga hutchinsonii* (YP_678271), and two additional short-chain dehydrogenases (SDR) from *Pseudomonas syringae* (YP_235378) and *Exiguobacterium sibiricum* (YP_001815303).

Detailed analysis of the metagenomic library revealed two clones out of 13 with identical open reading frames for a short-chain dehydrogenase/reductase showing reproducible activity on D-glucose. The remaining 11 clones obtained from the high-throughput screening exhibited no reproducible activity on glucose caused by possible variations in cultivation or cell disruption during the high-throughput screening process in 96 well plates. The analysis of the gene sequence of the cloned metagenomic DNA-fragment from clone 1E5 revealed a total insert size of 7561 bp containing 6 open reading frames including a sequence for a putative oxidoreductase with similarities to glucose dehydrogenases. The 986 nucleotides encoded for a putative protein of 328 amino acids showing 87% sequence similarity in to a putative oxidoreductase from the genome sequence of *Cronobacter sakazakii* (YP_001436414). The predicted molecular weight of the GDH1E5 was 35.54 kDa with an isoelectric point (pI) of 6.09. Compared with the well characterized glucose hydrolyzing SDR enzymes from *Bacillus megaterium* (P10528) and *B. subtilis* (P12310), GDH1E5 showed sequence similarities of 33 and 35%, respectively. Multiple sequence alignments using the described glucose dehydrogenases from *Bacillus*-species, two additional glucose dehydrogenases from *Burkholderia xenovorans* (YP_554854) and *Cytophaga hutchinsonii* (YP_678271) and two short-chain dehydrogenases from *Pseudomonas syringae* (YP_235378) and *Exiguobacterium sibiricum* assigned the GDH1E5 to the superfamily of SDR enzymes. The most conserved motifs are within the N-terminal sequence of the Rossmann-fold participating in the binding of dinucleotids on the one hand as well as the active site residues on the other hand. In the binding of the co-factor, a glycine rich phosphate-binding loop with the consensus sequence TGx_3_GxG is involved, which connects the C-terminus of β1-sheet with the N-terminus of the αA-helix. In addition to many other FAD or NAD(P) dependent SDR enzymes, a valine or isoleucine at the C-terminus of the β1-sheet is characteristic. A conserved catalytic tetrade is composed of N_194_, S_220_, Y_233_, K_237_ residues, which is present in many studied “classical” SDR enzymes (Figure 1). A homology based overall structure modeling of GDH1E5 with its predicted co-factor molecule NADP^+^ is given as Figure S1.

### Expression of the GDH1E5

**Figure 2:**
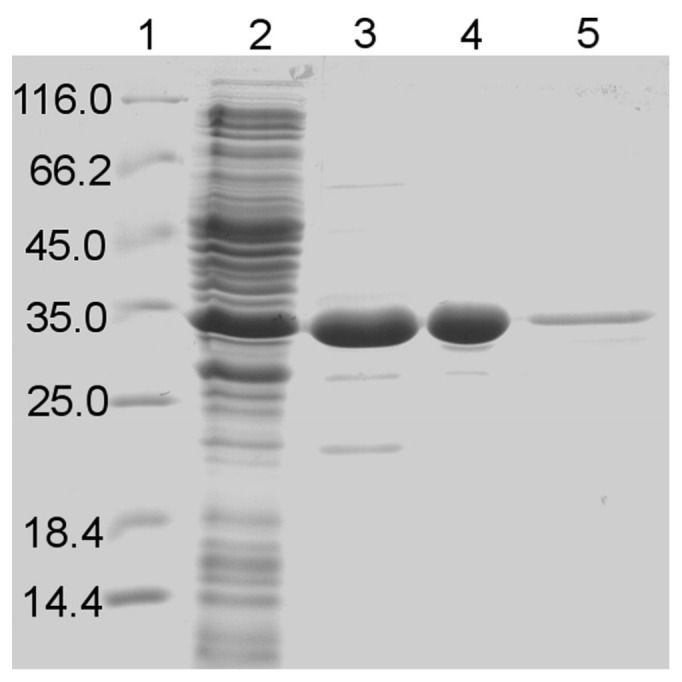
SDS-polyacrylamide gel electrophoresis (12%) of the purification of GDH1E5. The purification of GDH1E5 was examined after each purification step by SDS-PAGE. Lane 1: Protein standards. Lane 2: Crude extract. Lane 3: Hydrophobic interaction chromatography. Lane 4: Gel filtration. Lane 5: Immobilized metal ion affinity chromatography.

The identified gene sequence was amplified by PCR and subcloned into *E. coli* expression host. The recombinant GDH1E5 was produced with a N-terminal (His)_6_-tag for purification using the pQE-80L vector system. Initial purification via immobilized metal ion chromatography resulted in poor recovery of the recombinant protein. Alternatively the enzyme was purified via hydrophobic interaction chromatography (HIC), followed by gel filtration and a final polishing step via IMAC. The corresponding SDS-PAGE and the purification table are shown in Figure 2 and Table 1.

**Table 1:** Purification table of the GDH1E5.

Purification step	Total protein [mg]^a^	Total activity [U]^b^	Specific activity [U/mg]	Yield [%]	Purification factor
crude extract	262.2	725.0	2.8	100.0	1.0
HIC	21.6	513.0	17.8	70.8	6.4
Gel filtration	1.9	173.9	91.5	24.0	32.7
Ni^2+^-NTA	0.1	11.1	92.1	1.5	32.9

^a^ Total protein was determined after cell disruption in the soluble fraction by the method of Bradford.

^b^ Total activity was determined using *p*-nitrosoaniline BM53.0861 as electron acceptor at 35°C and pH 6.0.

**Figure 3:**
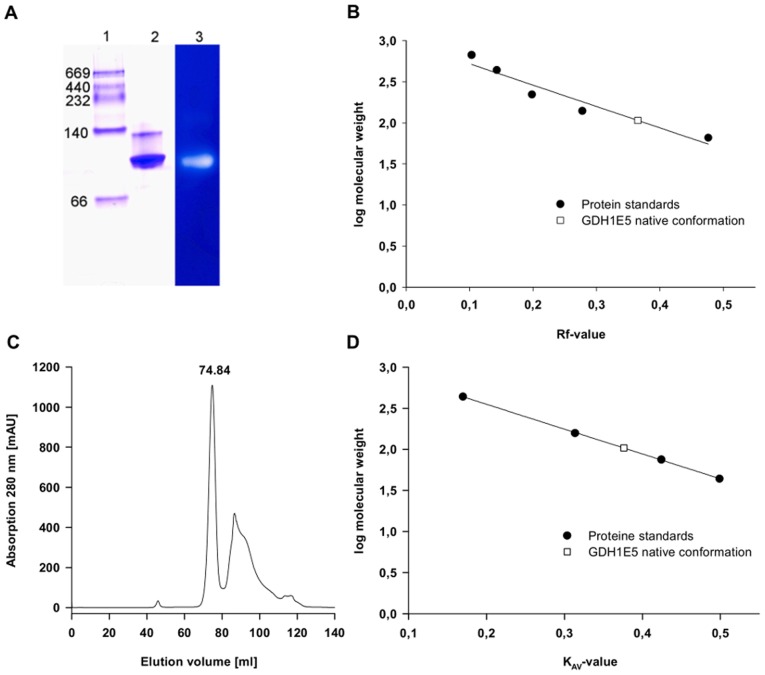
Molecular weight detection of native GDH1E5. A: Native polyacrylamide gelelectrophoresis of the size exclusion chromatography step in combination with a glucose dehydrogenase activity staining. Lane 1: marker proteins: Thyroglobulin (669 kDa), Catalase (232 kDa), Lactate-Dehydrogenase (140 kDa) and Albumin (66 kDa). Lane 2: GDH1E5 after size exclusion chromatography step stained with Coomassie. Lane 3: Activity staining of the native PAGE using glucose as substrate and DCPIP as colorimetric indicator and electron acceptor. Glucose-Dehydrogenase activity is indicated by a clear halo in regards to the decolorization of reduced DCPIP. B: Plot of the retention value Rf of the marker proteins and GDH1E5 versus the logarithm of their regarding molecular weight. C: Chromatogram of the purification step of GDH1E5 via size exclusion chromatography using HiLoad 16/60 Superdex 200 prep grade. D: Plot of partition coefficient K_AV_ from the derived elution volumes of the marker proteins (Ferritin 440 kDa, Aldolase 158 kDa, Conalbumin 75 kDa, Ovalbumin 44 kDa) and GDH1E5 versus the logarithm of their regarding molecular weight.

The molecular weight of a single GDH1E5 subunit was determined experimentally to be 37.7 kDa by SDS-PAGE (Figure 1). This value corresponds with the calculated molecular weight for a single subunit of 37.2 kDa including the N-terminal (His)_6_-tag. The molecular weight of the native enzyme was estimated to be 103.6 kDa by size exclusion chromatography and 107.0 kDa by native polyacrylamide gel electrophoresis. The average molecular weight of the native GDH1E5 was determined to be 105.4 kDa ±2.5 indicating that the native enzyme acts as a trimeric proteine of identical subunits (Figure 3).

### Absorbance Spectra

**Figure 4:**
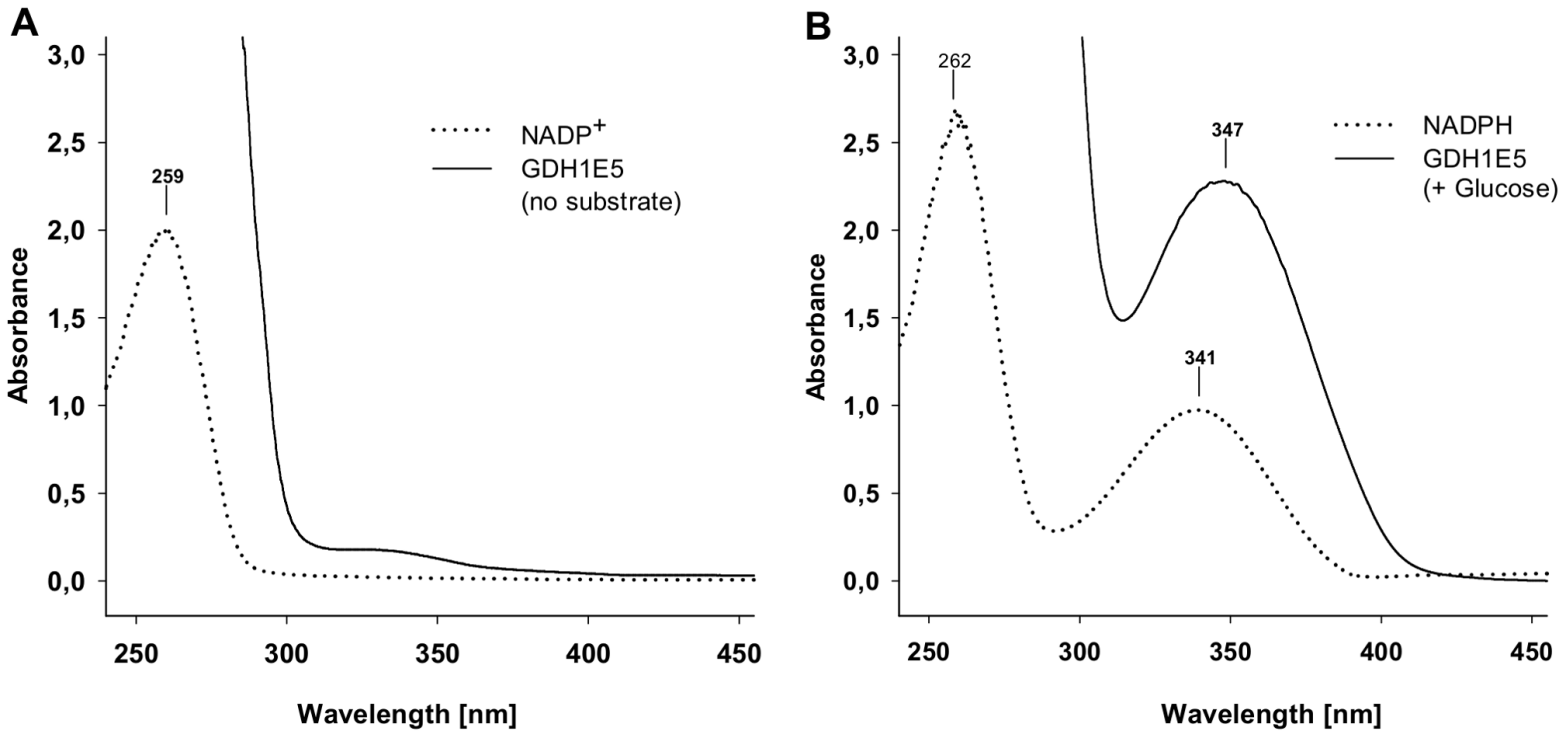
Absorbance spectra of GDH1E5 in absence and presence of glucose. A: Absorbance spectrum of purified GDH1E5 in the absence of substrate and external electron acceptors compared to the absorbance spectrum of the oxidized co-factor molecule NADP^+^. B: Absorbance spectrum of purified GDH1E5 in the presence of glucose and absence of external electron acceptors compared to the absorbance spectrum of the reduced co-factor molecule NADPH.

The sequence analysis of the GDH1E5 revealed the presence of a N-terminal Rossmann-fold participating in the binding of dinucleotids like NAD(P)^+^ to the enzyme. This well-known co-factor binding domain fulfills the selective binding of an essential oxidized co-factor molecule. The enzymatic oxidation of the substrate accompanying with the reduction of the attached co-factor followed by an ordered release of the reduced co-factor molecule. In the case of various described NAD(P)^+^-dependent enzymes, this ordered release of NAD(P)H from the enzyme can be monitored by an increase of absorbance at 340 nm representing the formation of free NAD(P)H. The existence of oxidized co-factor molecules NAD^+^ and NADP^+^ in the enzyme preparation of GDH1E5 showed in the presence of the enzymes substrate glucose no changes in absorbance in the colorimetric assays at 340 nm. However, the enzyme was able to transfer electrons from the oxidation of glucose to artificial electron acceptors like BM53.0861 and DCPIP in the complete absence of external co-factor molecules (Table 2). This gives rise to a bound Co-factor molecule which stays attached to the enzyme. Figure 4 shows the absorbance spectra of purified GDH1E5 in absence (A) and presence (B) of glucose without any artificial electron acceptors compared to the absorbance spectra of pure NADP^+^ and NADPH. In the presence of glucose, a characteristic peak with its absorbance maximum at 347 nm appears (B), representing the attached Co-factor in the reduced state. Due to the lack of artificial electron acceptors in the enzyme preparation, the co-factor retained in its reduced state creating the characteristic absorbance peak.

**Table 2:** Evaluation of various enzymatic assays for glucose dehydrogenase activity of GDH1E5.

Co-factor	external electron acceptor	Enzyme activity [U/mg]^a^
NAD^+^	–	no activity
NADP^+^	–	no activity
–	*p*-nitrosoaniline(BM53.0861)	91.5 (±0.4)
–	DCPIP	48.0 (±0.9)

^a^ All data represent the average of triplicate determinations ± standard derivation.

All enzyme assays were carried out using purified GDH1E5 over 5 min at 35°C and 75 mM glucose as a substrate. NAD(P)^+^ was used in concentrations of 5 mM respectively. The formation of reduced NAD(P)H was followed by an increase in absorbance at 340 nm. The enzymatic reduction of *p*-nitrosoaniline BM53.0861 and DCPIP was followed by an increase in absorbance at 620 nm for the nitrosoaniline and a decrease in absorbance at 600 nm for the dichloroindophenol.

### Substrate Specificity

The activity of the glucose dehydrogenase was investigated using various monomeric and dimeric sugars as substrate (Table 3). The determined dehydrogenase activity was highest with the non-phosphorylated aldohexose D-glucose. The enzyme showed almost comparatively high activity with the C-6 phosphorylated D-glucose (rel. activity 98.7%). By using the C-1 phosphorylated variant of D-glucose as a substrate, the enzyme was nearly inactive (rel. activity 1%). Compared to glucose, the enzyme showed a relative activity of 96.2% towards D-galactose, the C-4 epimere of D-glucose. A change in the configuration of the second chiral center of D-glucose leads to the C-2 epimere D-mannose. With this stereoisomer the enzyme showed low activity of 9.1%. The replacement of 2-hydroxyl group by a hydrogen atom led to a slight increase of activity (rel. activity 30.7%). The relative activity towards fructose was 1.1%. In contrast C-6 phosphorylated fructose leads to an increased activity. Besides activity on different aldohexoses the enzyme also shows 99.2% activity on the pentose D-xylose. From the tested disaccharides, lactose and maltose are hydrolyzed with a relative activity of 70.5% and 32.5% respectively.

**Table 3:** Substrate specificity of the GDH1E5.

Substrate	Specific activity (U/mg)^a^	Rel. activity (%)^b^
D-glucose	91.5 (±0.4)	100.0
D-xylose	90.8 (±3.3)	99.2
glucose-6-phosphate	90.3 (±3.9)	98.7
D-galactose	88.0 (±2.4)	96.2
lactose	64.5 (±0.6)	70.5
maltose	29.7 (±0.6)	32.5
2-deoxyglucose	28.1 (±0.9)	30.7
fructose-6-phosphate	10.2 (±0.8)	11.1
D-mannose	8.3 (±0.7)	9.1
sorbitol	2.6 (±0.4)	2.8
fructose	1.0 (±0.1)	1.1
glucose-1-phosphate	0.9 (±0.2)	1.0

^a^ All data represent the average of triplicate determinations ± standard derivation using *p*-nitrosoaniline BM53.0861 as electron acceptor at 35°C and pH 6.0. All substrates were tested at 75 mM concentration.

^b^ rel. activity of all substrates calculated in relation to the activity towards glucose-6-phosphate.

### Kinetic Constants

The kinetic constants of the GDH 1E5 for glucose-6-phosphate, D-glucose, D-xylose and maltose were determined at 37°C with *p*-nitrosoaniline BM53.0861 as electron acceptor at pH 6.0 (Table 4). The enzyme showed a high affinity to the substrates D-glucose (1.7 mM), D-xylose (1.5 mM) and glucose-6-phosphate (1.8 mM). GDH1E5 showed a 45 fold higher *K*_m_-value towards maltose in comparison to the monosaccharides. This indicates a significant lower affinity to the corresponding disaccharide (76.4 mM). The turnover number (k_cat_) was calculated using the experimentally-determined molecular weight of the native homotrimeric enzyme (105.4 kDa). The maximum turnover number was achieved with D-glucose (160.7 s^−1^) followed by D-xylose (159.5 s^−1^) and glucose-6-phosphate (158.6 s^−1^). The catalytic efficiency for the tested monosaccharides ranged from 90.1 s^−1^ mM^−1^ for glucose-6-phosphate to 92.9 s^−1^ mM^−1^ for D-glucose to 110.0 s^−1^ mM^−1^ for D-xylose. Resulting from the low affinity and turnover number for the disaccharide the catalytic efficiency for maltose was rather low.

**Table 4:** Kinetic constants of the GDH1E5 with various substrates.

Substrate	*K*_m_ [mM]^a^	k_cat_ [s^−1^]	k_cat_/*K*_m_ [s^−1^ mM^−1^]
D-glucose	1.7	160.7	92.9
D-xylose	1.5	159.5	110.0
glucose-6-phosphate	1.8	158.6	90.1
maltose	76.4	52.2	0.7

All data represent the average of triplicate determinations using *p*-nitrosoaniline BM53.0861 as electron acceptor at 35°C and pH 6.0.

^a^ Equivalent to the Michaelis-Menten constant. Variable glucose-6-phosphat concentration: 0.1–150 mM; Variable D-glucose and D-xylose concentration: 0.1–100 mM; Variable maltose concentration: 0.5–300 mM.

### Effect of Temperature on Activity and Stability of the GDH1E5

**Figure 5:**
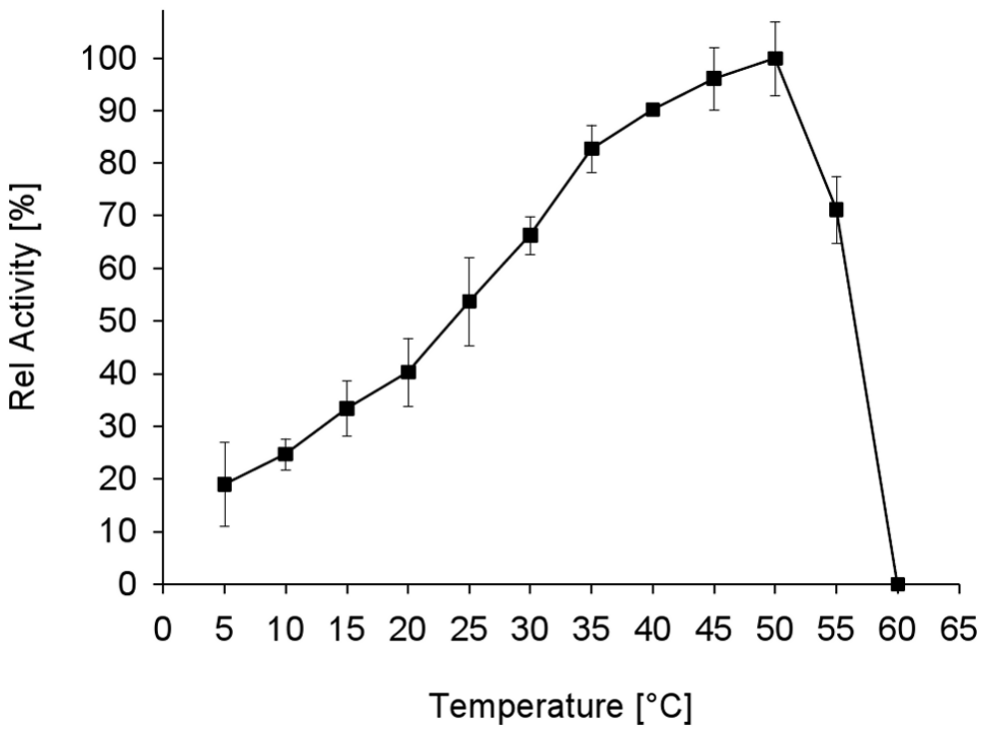
The effect of temperature on the activity of GDH1E5. The enzyme activity was assayed at various temperatures in the range of 5 to 60°C. All solutions were preincubated at the corresponding temperature and the enzymatic activity was assayed for 5 min at the respective temperature using DCPIP as electron acceptor. All data represent the average of triplicate determinations ± standard derivation.

The effect of temperature on the activity and the stability of the recombinant enzyme was investigated. The influence of temperature on activity using DCPIP and D-glucose as a substrate showed activity over a broad temperature range (Figure 5). The GDH1E5 showed maximum activity at a temperature of 50°C. At a temperature of 5°C the enzyme remained 20% of its maximum activity. At elevated temperatures of 55 and 60°C a rapid inactivation of the enzyme was observed.

**Figure 6:**
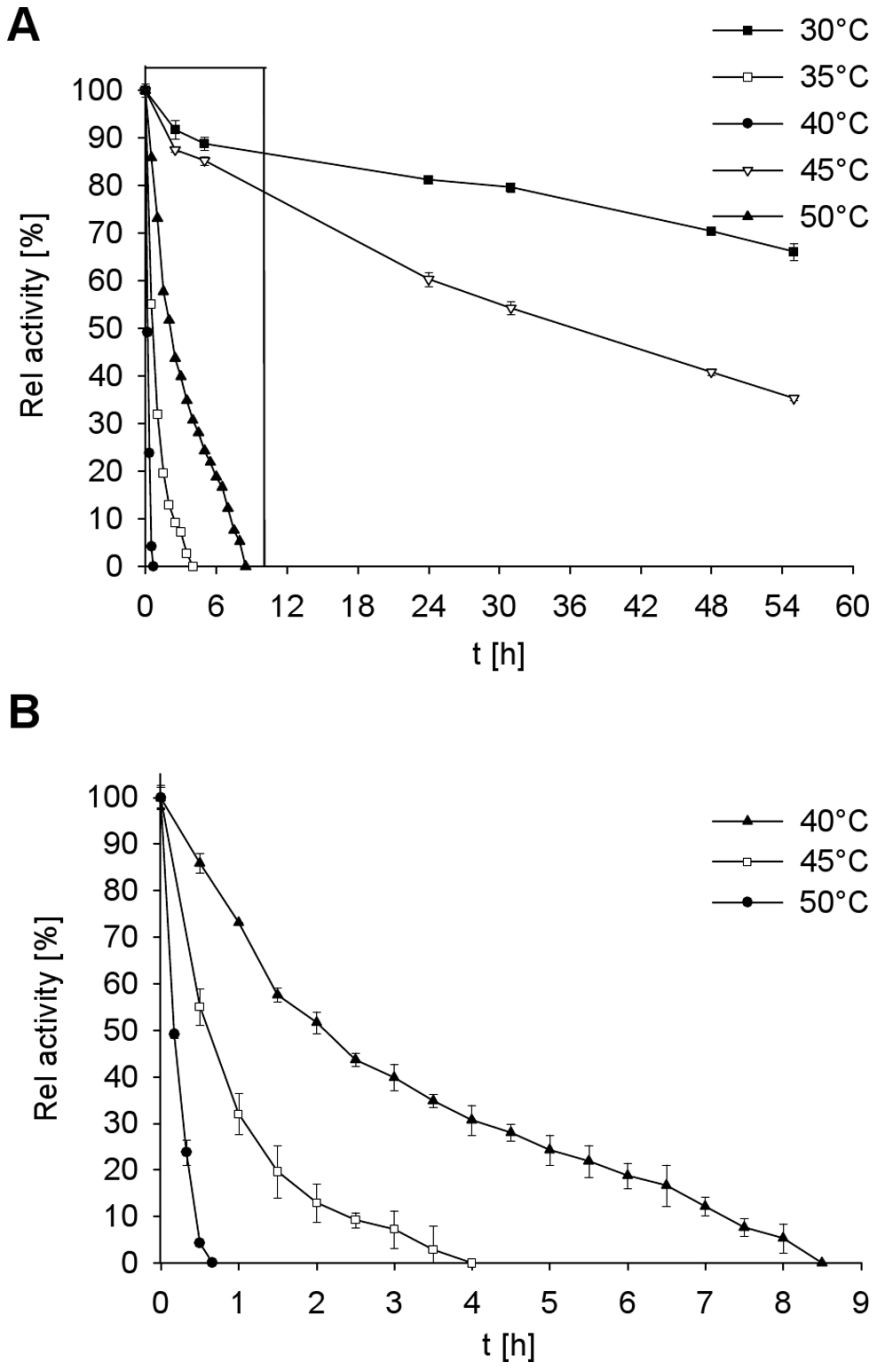
Influence of different temperatures on the stability of GDH1E5. The residual activity of the enzyme was assayed after incubation at various temperatures in the range of 30 to 50°C. The residual activity was determined for 5 min at 35°C using D-glucose as substrate and DCPIP as electron acceptor. All data represent the average of triplicate determinations ± standard derivation. A: Thermal stability of the GDH1E5 over a span of time from 0–55 h. B: Zoomed view of the stability of the GDH1E5 at temperatures from 40 to 50°C over a span of 9 h (boxed area in A).

The temperature dependent stability of the GDH1E5 was examined at temperatures in the range of 30 to 50°C. After incubation of the enzyme for 55 h at 30°C residual activity was 66%. The half-life of the GDH1E5 at 35°C was 36 h. A temperature shift to 40°C resulted in a decrease in temperature stability compared to 35°C. At 40°C the half-life of the enzyme was reduced to two hours. After 8.5 h a complete inactivation of the GDH1E5 at 40°C was observed. The thermostability at 45 and 50°C was rather low. At 45°C the GDH1E5 was completely inactivated after 4 h and at 50°C after 40 min (Figure 6).

**Figure 7:**
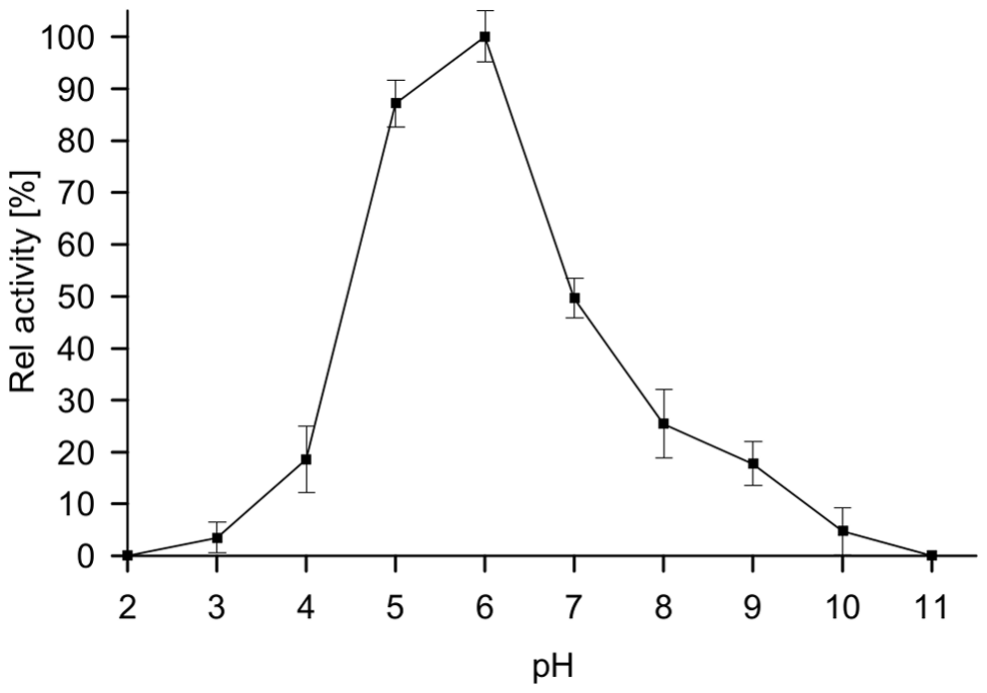
pH profile of GDH 1E5. The enzyme activity was assayed at various pH values in Britton & Robinson universal buffer in the range of pH 2–11 using DCPIP as electron acceptor. The enzymatic activity was assayed for 5 min at 35°C and the according pH using DCPIP as electron acceptor. All data represent the average of triplicate determinations ± standard derivation.

The optimal pH was determined in Britton & Robinson universal buffer in the range of pH 2–11 using DCPIP as electron acceptor. The enzyme showed maximum activity on D-glucose at a pH of 6.0. The enzyme lost 50% of its maximum activity at pH 7.0. The GDH1E5 was completely inactivated at both an alkaline pH of 11.0 and an acidic pH of 2.0 (Figure 7).

**Figure 8:**
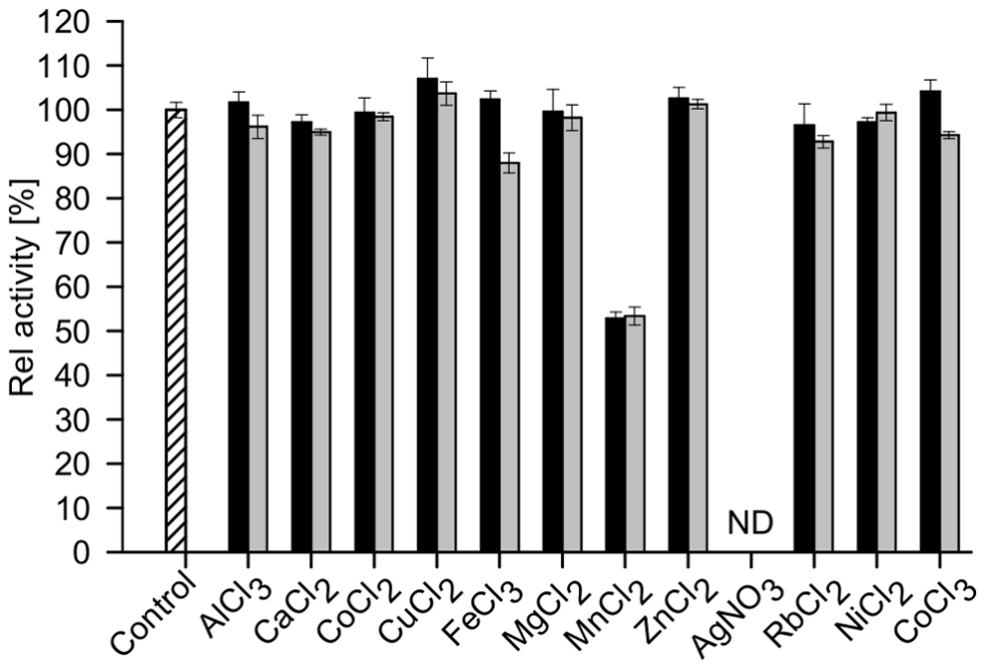
Effect of various metal ions on GDH1E5. The influence of di- and trivalent cations on enzyme activity was investigated using D-glucose as substrate and DCPIP as electron acceptor. Residual activity was determined after enzyme preincubation for 30 min in the presence of 1 (black bars) and 5 mM (grey bars) of the corresponding ion in citrate buffer (pH 6.0). Afterwards enzymatic activity was determined in the presence of the cations using DCPIP at 35°C.

**Figure 9:**
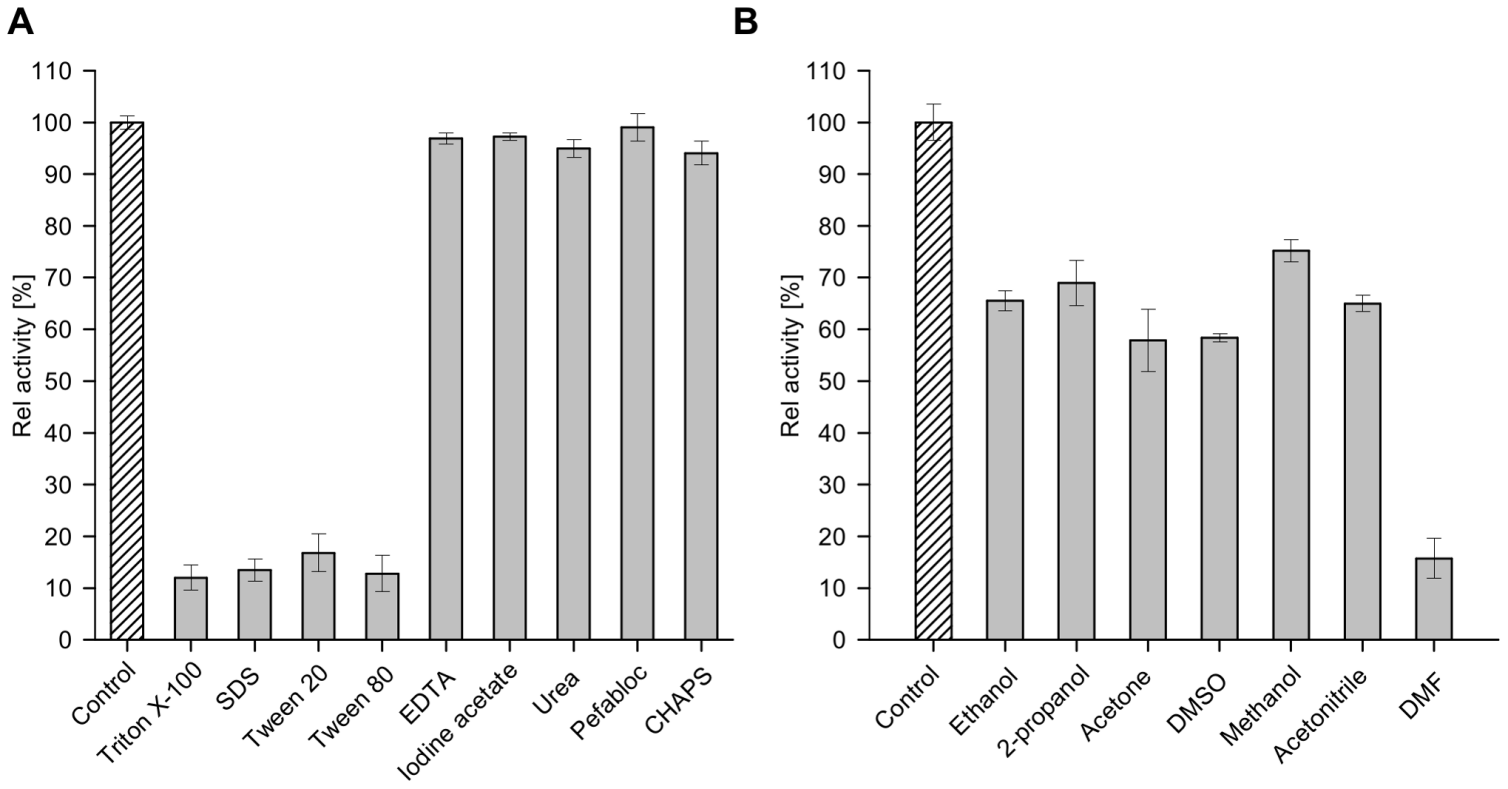
Effect of various detergents and organic solvents on GDH1E5. The influence of detergents (A) and organic solvents with respective concentrations of 1% (w/v, v/v) and 10% (v/v) was investigated for enzyme activity using D-glucose as substrate and DCPIP as electron acceptor. Residual activity was determined after preincubation of the enzyme for 30 min in the presence of the corresponding detergents and organic solvents in citrate buffer (pH 6.0) and sodium phosphate buffer (pH 6.0) respectively. Afterwards enzymatic activity was determined in the presence of the detergents and organic solvents using DCPIP at 35°C.

The effect of different di- and trivalent cations on enzyme activity was investigated using D-glucose as substrate and DCPIP as electron acceptor. All tested metal ions except Mn^2+^ and Ag^3+^, showed no significant inhibitory or activating effect on enzyme activity (Figure 8). At concentrations of 1 and 5 mM of Mn^2+^ ions the enzyme lost 50% of its activity. In the presence of Ag^3+^ ions no residual activity of GDH1E5 could be detected. The detergents SDS and Triton® X-100, as well as the emulsifiers Tween® 20 and 80 had a strong influence on the enzyme (Remaining activity 10–20%). The GDH1E5 retained 80–90% of its activity in the presence of these detergents. Other tested reagents like ethylendiaminetetraacetic acid (EDTA), iodine acetate, urea, Pefabloc and CHAPS did not influence the enzymatic activity (Figure 9A). Furthermore, the effect of several organic solvents like ethanol, isopropanol, acetone, methanol, acetonitrile, dimethyl formamide (DMF) and dimethly sulfoxide (DMSO) in a final concentration of 10% (v/v) was examined. The GDH1E5 showed residual activity of 20% in the presence of 10% DMF. The presence of all the other solvents resulted in relative activities between 60 and 80% (Figure 9B).

## Discussion

In the aim of finding a novel glucose hydrolyzing enzyme, we focused our screening on environmental metagenomic libraries expressed in *E. coli*. In this approach we were able to isolate an unidentified enzyme via activity based high-throughput screening from uncultivable microorganisms. The metagenomic sequence showed highest similarity to a non-characterized genome sequence of *Cronobacter sakazakii*, a species related to the family of *Enterobacteriaceae*. From different isolation experiments, it is well-known that various representatives of the *Enterobacteriaceae* are present in several environmental samples of moderate temperatures, whether water or soil [29]. The source of the GDH1E5 could be a member of the family of *Enterobacteriaceae* or a close relative of this family.

The isolated enzyme showed highly conserved sequence motifs and the typical secondary structure of alternating β-sheets and α-helices of the Rossmann-fold for the binding of dinucleotides. The identified sequences, the strand topology with the relative order 321456, as well as the absorbance spectra of the purified enzyme, indicate that NAD(P) is more suitable than FAD as co-factor [30]. Moreover, at position 13 C-terminal of the conserved sequence motive V/ITGxxx[S]GxGxxxA the amino acid asparagine is present. In many NAD-dependent enzymes a characteristic aspartate is located at this position. This replacement of the conserved aspartate by an asparagine is significant for the mechanism of discriminating between NAD and NADP binding sites in many NAD-dependent enzymes [30]. The active site residues N_194_S_220_Y_233_K_237_ form the catalytic tetrate and classify the GDH1E5 under the consideration of the conserved cofactor binding site into the subfamily of “classical” SDR [15], [12]. The conserved N_259_ residue located in strand β-6 embedded in the short conserved structural element consisting of the amino acids IRVN is also present in the characterized enzyme. As described by Filling *et al*. the Asn side chain plays an important role in the formation of an essential hydrogen bonding network which is necessary for the catalytic mechanism in SDR enzymes. Mutagenesis experiments at this position were shown to result in a complete loss of enzymatic function [31].

In most of these cases an ordered binding of NAD(P)^+^ and the substrate is involved, followed by the ordered release of the oxidized educt and the reduced co-factor NAD(P)H [21], . In general, the formation of free NAD(P)H gained by the enzymatic reaction of many NAD(P)-dependent enzymes can be followed in a colorimetric assay by an increase of absorbance at a wavelength of 340 nm. In the case of GDH1E5 no release of the reduced co-factor after the oxidation of the substrate could be observed, indicating that the reduced NAD(P)H remains attached to the enzyme (Figure 4). The addition of an external electron acceptor like *p*-nitrosoanilines or DCPIP is therefore essential for the enzymatic activity to recycle the reduced co-factor associated with the enzyme (Figure 4). The use of expensive co-factor molecules or the implementation of complex co-factor recycling systems to maintain the enzymatic activity of GDH1E5 can be neglected by the use of specific mediators like BM 53.0861 related to blood glucose test stripes.

The results indicate that the transfer of electrons from NAD(P)H to the external electron acceptor is the speed limiting step in the reaction. The maximum detected activity was achieved by using *p*-nitrosoaniline BM 53.0861 followed by DCPIP as electron acceptors (Table 2). The overall high hydrophobicity of 52.4% of the amino acid residues of the GDH1E5 could be due to the strong interaction of the enzyme with its co-factor in aqueous solutions.

The recombinant GDH1E5 is specific for the hexoses glucose, glucose-6-phosphate, galactose and the pentose xylose. For the phosphorylated glucose the position of the phosphate group is of great importance for the catalytic activity of the enzyme. The enzyme lacks activity if the phosphate group is located on the C-1 position of glucose. This indicates that the orientation of the substrate molecule to the active site is of particular significance for the enzymatic activity. In addition, the stereochemical configuration of the hydroxyl group of the C2-atom seems to influence the catalysis of the substrate by the GDH1E5 as well. Towards mannose, the enzyme showed a relative activity of less than 10%, compared to D-glucose. The phenomenon of the discrimination between different sugar isomers was also observed in the aldohexose dehydrogenase from *Gluconobacter cerinus* and *Pseudomonas* sp. [34], [35]. Crystallization experiments with the aldohexose dehydrogenase from *Thermoplasma acidophilum* revealed structural insights into the substrate selectivity of the enzyme. It was observed that the conformation of a glutamate side-chain at the substrate binding pocket is responsible for the discrimination between the two C2-isomers mannose and glucose by the enzyme [36].

The highest specific activity towards the tested dimeric sugars was shown to be with lactose (rel. activity 70.5%). Towards all the other tested dimeric sugars like maltose, sucrose and fructose the GDH1E5 showed relative activities between 0–32.5%. The specific activities of the recombinant GDH1E5 are similar to the characterized glucose dehydrogenase from the halophilic archaeon *Haloferax mediterranei* [37]. Compared to characterized glucose dehydrogenases from *Bacillus* species, the metagenomic GDH1E5 showed a broader substrate range. The main difference between the GDH1E5 and the GDH from *Bacillus megaterium* is the significant lower activity of GDH1E5 towards 2-deoxyglucose and the increased activity of the GDH1E5 on the monosaccharides xylose, mannose and galactose [38], [39]. Different characterized glucose hydrolyzing enzymes showed either high activity on glucose with respective low activity on glucose-6-phosphate or vice versa [40], [41].

The GDH1E5 exhibits particularly high affinities to the monosaccharides glucose, glucose-6-phosphate, and xylose compared to other characterized glucose dehydrogenases [42]–[44], [39]. The *K*_m_-value of the disaccharide maltose was remarkably higher with a factor of 45 compared to the tested monosaccharides. As expected, the catalytic efficiency of the enzyme against maltose is rather low, resulting from the comparatively high *K*_m_-value. This could be due to the size of maltose which could result in steric hindrance and a consequent poor accessibility of the dimeric sugar to the catalytic site of the enzyme.

The temperature profile of GDH1E5 showed enzymatic activity over a broad temperature range from 5 to 55°C. However, the GDH1E5 revealed low stability at 50°C. This indicates that the gene was most probably derived from a mesophilic uncultured host. The optimum pH for maximum substrate conversion of GDH1E5 was determined to be in a slight acidic milieu around pH 6. For other characterized glucose hydrolyzing enzymes from bacterial and fungal sources an optimal pH in a neutral to slightly alkaline pH is described [42], [44]–[48].

In conclusion, we were able to establish a high-throughput screening and identify a novel glucose hydrolyzing enzyme belonging to the family of SDR enzymes from a metagenomic library derived from a hay infusion. The low *K*_m_ value towards glucose, strong association of the co-factor molecule, high stability, and ability to transfer electrons from the oxidation of glucose to external electron acceptors related to test stripes for blood glucose determination makes this enzyme suitable for the application in glucose sensors.

## Supporting Information

Figure S1 **Homology based modeling of the 3D structure of GDH1E5.** A: Overall structure modeling of GDH1E5 with its associated co-factor molecule NADP^+^. α-helices are depicted in orange and β-sheets in blue. The active site amino acids are highlighted in green. The characteristic Rossmann-fold with alternating β-sheets inside, flanked by three α-helices from each site is involved in co-factor binding. B: Insight view of the predicted amino acid residues involved in NADP^+^-binding. Potential H-bounds are denoted in dotted lines. The amino acid residues of the catalytic tetrad Asn_194_, Ser_220_, Lys_237_, Tyr_233_ are highlighted in green again. All 3D structures were modeled using the SWISS-MODEL workspace and visualized by the PdbViewer [49].
(TIF)
