# Supplementary figures and images for: Isolation and Biochemical Characterization of a Glucose Dehydrogenase from a Hay Infusion Metagenome
Source: PLoS One. 2014 Jan 14;9(1):e85844. doi: 10.1371/journal.pone.0085844 (PMC3891874; doi:10.1371/journal.pone.0085844)

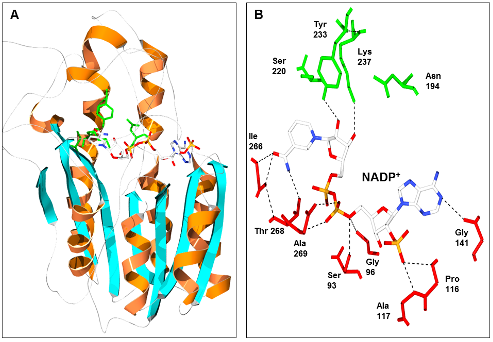

Supplement: Figure S1 — Homology based modeling of the 3D structure of GDH1E5. A: Overall structure modeling of GDH1E5 with its associated co-factor molecule NADP+. α-helices are depicted in orange and β-sheets in blue. The active site amino acids are highlighted in green. The characteristic Rossmann-fold with alternating β-sheets inside, flanked by three α-helices from each site is involved in co-factor binding. B: Insight view of the predicted amino acid residues involved in NADP+-binding. Potential H-bounds are denoted in dotted lines. The amino acid residues of the catalytic tetrad Asn194, Ser220, Lys237, Tyr233 are highlighted in green again. All 3D structures were modeled using the SWISS-MODEL workspace and visualized by the PdbViewer [49]. (TIF) [file pone.0085844.s001.tif]
